# Effects of 16-week tai chi practice on sit-to-stand performance and lower-limb neuromuscular control strategies in community-dwelling older adults: a randomized controlled trial

**DOI:** 10.3389/fphys.2025.1681591

**Published:** 2025-10-15

**Authors:** Lyu Mozhu, Li Jiayu

**Affiliations:** ^1^ School of Martial Arts and Dance, Shenyang Sport University, Shenyang, Liaoning, China; ^2^ School of Martial Arts, Henan University, Zhengzhou, Henan, China

**Keywords:** aging, sit-to-stand transition, postural stability, neuromuscular control, tai chi exercise

## Abstract

**Background:**

The ability to perform sit-to-stand (STS) transitions is a fundamental marker of functional independence in older adults, and age-related declines in STS performance are strongly linked to increased fall risk and compromised quality of life. While mind–body exercises like Tai Chi have shown promise in enhancing physical function in this population, the specific neuromuscular mechanisms through which Tai Chi improves STS performance—particularly how it modulates biomechanical patterns and muscle control during this critical movement—remain poorly understood.

**Objective:**

This study aimed to investigate the effects of a 16-week, Yang-style Tai Chi programme on STS performance and lower limb neuromuscular control strategies underpinning any improvements in older adults. Methods: Sixty community-dwelling older adults (60–75 years) stratified by sex and baseline STS time. The 32 older adults were recruited as the experimental group (Tai Chi practitioners), and 28 as the control group (maintaining habitual lifestyle). The experimental group underwent 16 weeks of Tai Chi practice (5 sessions/week, 40 min/session), while the control group maintained their original daily routines. Before and after the intervention, the following parameters were measured during STS transitions: center of pressure (COP), temporal parameters, and surface electromyography (sEMG) signals of relevant lower limb muscles.

**Results:**

Data indicated that with increasing age, older adults exhibited decreased physical activity levels and impaired STS ability. After 16 weeks of Tai Chi practice, the experimental group showed significant improvements in STS ability, with marked reductions in reaction time, standing phase duration, and total time for Five Times Sit-to-Stand Test (FTSST). During STS tasks, the sample entropy of COP in the experimental group significantly decreased, accompanied by reduced sEMG amplitudes of the biceps femoris, rectus femoris, and tibialis anterior.

**Conclusion:**

This study demonstrated that aging is associated with declines in physical activity, reaction capacity, and STS ability in older adults. Tai Chi improves STS performance through greater neuromuscular efficiency and reduced postural sway, providing a mechanistic rationale for its integration into fall-prevention programs for older adults.

## Introduction

With the acceleration of global aging, the decline in functional activity capacity among older adults has become a critical public health concern. The ability to perform sit-to-stand (STS) transitions is a core indicator for evaluating lower limb muscle strength, dynamic balance, and functional independence in older adults (Xue et al., 2021). Notably, the STS task not only assesses lower limb strength but also captures essential aspects of dynamic balance control during the transition from sitting to standing—a phase particularly vulnerable to instability and falls in the elderly (Xue et al., 2021). While the Timed Up and Go (TUG) test is also widely used to assess similar constructs such as mobility and dynamic balance, the STS task was selected for this study due to its higher sensitivity in isolating and quantifying lower limb strength and power—a key factor in functional decline and fall risk in the elderly. STS performance provides a more direct measure of lower extremity muscle function without the confounding influence of gait and turning elements inherent in the TUG (Goldberg et al., 2012; Millor et al., 2013). Studies have shown that older adults with difficulty in STS often experience muscle atrophy and reduced balance, which not only impair daily living independence but also increase fall risk (Ejupi et al., 2015; Zhang et al., 2013; Atrsaei et al., 2022), posing serious threats to their health and quality of life. Physiologically, STS requires coordinated activation of the nervous and musculoskeletal systems. Age-related declines in neurological function (e.g., slowed nerve conduction) and muscle strength/endurance directly impair STS performance (Singh and Natsume, 2023; Yamada and Demura, 2009). Identifying safe, effective exercise interventions and their underlying neuromuscular mechanisms is therefore critical.

Numerous studies have investigated exercise interventions to enhance lower limb muscle strength and motor function in older adults. Resistance training is well-established for improving lower limb muscle strength and endurance. Studies by Lai et al. (2021) and Lai et al. (2023) demonstrated its effectiveness in enhancing quadriceps and hip extensor strength, leading to reduced time in the Five Times Sit-to-Stand Test (FTSST). Similarly, a randomized controlled trial by Fiatarone et al. (1994) reported substantial improvements in knee extension strength and walking speed among old adults after high-intensity resistance training. A meta-analysis by Straight et al. (2016) further supports these findings, indicating that moderate-to-high intensity resistance training yields small-to-moderate improvements in muscle power in older adults. Although these studies show that resistance training improves STS performance, evidence specifically regarding its effects on dynamic balance control during the STS task remains limited (Straight et al., 2016; Lai et al., 2023). For instance, while resistance training improves muscle power, its focus is less on the integrated postural adjustments and weight-shifting coordination that are critical for balance during dynamic functional tasks like STS.

Aerobic exercise has also been explored for improving STS performance, but evidence points to limitations. A randomized controlled trial (RCT) in sedentary older adults (Zhang et al., 2024) found aerobic exercise less effective than combined aerobic-resistance training for STS performance and dual-task gait speed—though both improved lower limb strength and dynamic balance. Another RCT similarly showed aerobic exercise boosted cardiopulmonary function but only half the dynamic balance gains of resistance training (Takeshima et al., 2007). This discrepancy arises because STS and dynamic balance demand rapid lower limb muscle recruitment and coordination, while aerobic exercise (e.g., brisk walking) mainly enhances cardiopulmonary endurance and slow-twitch fibers, with minimal stimulation of muscle power, neural control, or the multisensory integration critical for balance. Thus, aerobic exercise benefits cardiopulmonary function but offers limited direct improvement for older adults’ STS ability.

In contrast, Tai Chi—a traditional Chinese mind-body exercise—complements the strengths of both resistance and aerobic training, offering unique advantages in geriatric rehabilitation. While resistance training effectively builds muscle strength and aerobic exercise improves cardiovascular endurance, Tai Chi integrates elements of strength, balance, flexibility, and neuromuscular control, making it particularly suitable for improving functional movements like STS in older adults. Regular practice has been shown to enhance lower limb muscle strength (Li et al., 2009), improves proprioception (Zhang et al., 2021) and joint flexibility (Ke et al., 2022), and boosts dynamic balance through center-of-mass transfer training (Tsang and Hui-Chan, 2008)—all of which collectively reduce fall risk (Jee et al., 2025; Zhou et al., 2016). Existing studies further confirm that Tai Chi enhances neuromuscular signal transmission efficiency (Gatts, 2008; Chen et al., 2021), and this enhancement provides a critical foundation for coordinated STS movements.

While previous studies have validated Tai Chi’s positive effects on older adults’ balance (Fan et al., 2025), the evidence remains limited: most focus on static balance tests (e.g., single-leg stance) or structured dynamic assessments (e.g., Y-balance scale). Notably, these traditional paradigms fail to capture the continuous movement characteristics of daily functional tasks (e.g., STS)—a gap that weakens their relevance to real-world mobility. To date, most research on relationships between Tai Chi and balance has centered on static tasks or gait, with no systematic investigation into dynamic STS. As a key functional movement, STS requires the integration of lower-limb neuromuscular control, postural adjustment, and cognitive reaction, and it is more closely linked to older adults’ daily functional independence than other balance-related tasks (Lord et al., 2002). Additionally, most existing studies are either observational or short-term interventions (<12 weeks), lacking long-term randomized controlled trials (RCTs) to verify sustained efficacy. Furthermore, current evidence primarily focuses on macro-functional improvements (e.g., overall balance, lower limb strength); few studies explore how Tai Chi modulates neuromuscular control strategies (e.g., muscle activation patterns) during STS itself. Despite meta-analytic evidence that mind–body exercise improves balance, no previous RCT has simultaneously examined biomechanical and electrophysiological adaptations underlying STS in older adults. Addressing this gap is critical for personalising exercise prescriptions and understanding the neural adaptations that drive functional gains.

This study examined the correlation between age and STS ability, followed by a 16-week randomized controlled trial. By integrating temporal parameters (reaction time, movement duration), COP sample entropy, and lower limb EMG characteristics, we systematically investigated the effects of Tai Chi on STS ability and neuromuscular control strategies in older adults, providing evidence-based support for its precise clinical application. This study provided two hypotheses: (H1) Compared with habitual activity, a 16-week Yang-style Tai Chi practice will significantly reduce the time required to complete the STS Test; and (H2) Concomitant with this functional improvement, Tai Chi practitioners will exhibit a significant decrease in COP sample entropy and lower-limb sEMG RMS amplitudes of the biceps femoris, rectus femoris, and tibialis anterior during the sit-to-stand transition, reflecting enhanced neuromuscular efficiency.

## Materials and methods

### Participants

Seventy-two older adults (60–75 years) were recruited from the community between October 2023 and February 2024. Inclusion criteria: age ≥60 years; ability to independently perform STS, stand, and walk short distances without assistive devices; no severe cardiovascular/cerebrovascular diseases (e.g., uncontrolled hypertension, coronary heart disease), musculoskeletal disorders (e.g., severe arthritis), neurological diseases (e.g., Parkinson’s disease), or balance impairments; Mini-Mental State Examination (MMSE) score ≥24 to ensure comprehension and cooperation; voluntary participation with signed informed consent. Exclusion criteria: recent fractures or surgeries (within 3 months); severe mental illness precluding cooperation; inability to complete the 16-week training program. Eligible participants (n = 72) were randomized into an experimental group and a control group (n = 36 each) by an independent researcher not involved in participant recruitment or outcome assessment using a computer-generated random number table (randomization sequence stored in sealed opaque envelopes). Outcome assessors (for STS timing, COP, and sEMG analysis) and data analysts were blinded to group allocation throughout the study. The experimental group received Tai Chi intervention, while the control group maintained their habitual lifestyle.

Sample size was calculated using GPower 3.1 (effect size f = 0.3, power = 0.8, α = 0.05) for a 2 × 2 repeated-measures ANOVA, estimating 21 participants per group to detect differences in STS total time (primary outcome). A 20% dropout rate was anticipated, leading to initial recruitment of 72 participants. During the trial, 4 participants dropped out from the experimental group and 8 from the control group, resulting in 32 participants in the experimental group (18 males, 14 females; 60–75 years) and 28 in the control group (16 males, 12 females; 60–74 years), totaling 60 participants. Baseline data (gender ratio, age, height, weight, physical activity level by the International Physical Activity Questionnaire (IPAQ), MMSE score) showed no significant differences between groups (*p* > 0.05) (Table 1). The study was approved by the Institutional Ethics Committee (No. 20231105).

**TABLE 1:** Comparison of baseline characteristics between groups (x̄±s).

Group	n	Gender (M/F)	Age (years)	Height (cm)	Weight (kg)	BMI (kg/m^2^)	Physical activity (MET-min/wk)	MMSE/Score
Tai Chi	32	18/14	66.9 ± 4.3	169.0 ± 7.2	65.3 ± 7.6	22.8 ± 1.5	578.4 ± 199.0	26.7 ± 4.9
Control	28	16/12	66.0 ± 3.7	168.5 ± 6.9	65.0 ± 7.7	22.8 ± 1.4	513.8 ± 154.8	25.3 ± 5.3
t/*x* ^*2*^ values		0.069	0.838	0.271	0.155	0.052	1.389	1.041
*p* values		0.944	0.405	0.787	0.877	0.959	0.170	0.303

## Intervention protocol

A randomized controlled design was adopted, with participants assigned to either the experimental group or the control group. The effects of group, time (pre-vs post-intervention), and movement phase on STS-related indices were observed. The experimental group underwent 16 weeks of Tai Chi practice: 5 sessions/week, 60 min/session, including 10 min of warm-up (e.g., joint mobilization), 40 min of Tai Chi practice (simplified 24-form Yang-style Tai Chi), including movements emphasizing lower-limb coordination and center-of-mass transfer, such as ‘Grasp Sparrow’s Tail’ (requiring knee flexion-extension) and ‘Cloud Hands’ (mediolateral weight shifting), which mimic STS-related motor patterns, and 10 min of cool-down (e.g., stretching, slow walking). Training was supervised by certified Tai Chi instructors (≥5 years of teaching experience) to ensure proper form. Intervention adherence was recorded as session attendance rate: the experimental group had a mean attendance rate of 92.3% ± 6.1% (range: 81%–100%), with absences due to temporary illness (n = 5) made up via 30-min individual catch-up sessions within 1 week. The control group maintained their habitual lifestyle, with weekly phone calls to monitor physical activity (via 7-day recall) by using the IPAQ (Craig et al., 2003) to quantify activity levels (MET-min/wk) across domains (leisure, household, transportation). Participants were instructed to avoid structured exercise (e.g., resistance training, Tai Chi, yoga) during the intervention, and adherence was confirmed via weekly self-report.

## Experimental procedures

Participants were instructed on standard STS movements: seated on a 43 cm-high chair with back straight against the chair, hands crossed over the chest, feet flat on a portable balance platform (HUMAC BALANCE, United States) and shoulder-width apart. A triaxial accelerometer (Boruikang, China) was placed at the waist (L4 spinous process, 2–3 cm above the midpoint of the bilateral iliac crests) (Marques et al., 2020) to: (1) detect the onset of trunk forward lean (marking reaction time start); (2) identify STS phases (standing: upward acceleration >0.5 g; sitting: downward acceleration < −0.5 g). Following an auditory cue delivered by the computer system, participants performed the Five Times Sit-to-Stand Test (FTSST). Each trial consisted of leaning forward, lifting the hips off the seat, straightening the back, and standing up until fully upright with knees extended. Participants then returned to a seated position. This sequence was repeated five times consecutively. Movements were required to be continuous and smooth, without arm assistance or external force. Participants completed two FTSST trials with a 5-min rest interval to avoid muscle fatigue. Formal testing steps were as following:

1. Surface electrodes were attached to the bilateral rectus femoris, tibialis anterior, and lateral gastrocnemius to collect EMG signals. An accelerometer was placed at the waist to identify movement phases.
2. After electrode placement, 3-5 practice trials were conducted to familiarize participants with the task and rhythm.
3. Following a 1–2 min rest, formal testing began. Upon the auditory cue, participants performed FTSST. Movements were monitored for compliance; non-compliant trials (e.g., excessive arm use, significant sway, incomplete standing/sitting) were repeated.
4. Upon completion of the 5th sitting movement, timing was stopped. The system automatically calculated total time for FTSST, standing duration, sitting duration, and reaction time based on accelerometer data.
5. After a 5-min rest, participants completed a second FTSST trial. Indices were averaged across the two trials.

### STS ability assessment

Based on literature (Marques et al., 2020; Hyun et al., 2021; Lord et al., 2002) and study-specific characteristics, temporal parameters during STS were defined as:

1. Reaction time: Interval from auditory cue to initial trunk forward lean (detected by accelerometer).
2. Standing time: Duration from hip-off-seat to full upright stance with knees extended and stable (accelerometer signal from onset spike to return to baseline).
3. Sitting time: Duration from upright stance to hip contact with the seat and stabilization (accelerometer signal from baseline to spike and return to baseline).
4. Total time for FTSST: Duration from the initial auditory cue to completion of the 5th sitting movement, including standing and sitting phases.

### Center of pressure (COP) assessment

During the pre-STS preparation phase, participants sat with bare feet on the balance platform. COP data during STS were collected at a sampling frequency of 100 Hz. COP data during standing and sitting phases were analyzed separately for mediolateral (COPx) and anteroposterior (COPy) directions. Sample entropy analysis was applied to COP data (Montesinos et al., 2018; Fischer et al., 2023), as it is suitable for analyzing complex biological signals: higher sample entropy indicates greater data variability, while lower values indicate improved stability during STS.

### Surface EMG signal collection and analysis

A portable EMG system (ME6000, Finland) was used to collect sEMG from lower limb muscles: bilateral biceps femoris, rectus femoris, tibialis anterior, and lateral gastrocnemius. Skin preparation included abrasion with sandpaper and cleaning with 70% isopropyl alcohol to reduce impedance. Bipolar electrodes were placed with a 2 cm inter-electrode distance, and reference electrodes were 2 cm from the recording electrodes, positioned over the muscle belly during maximal contraction. According to literature (Winter et al., 1994), the sEMG electrodes were placed as follows: (1) for the biceps femoris: along the midpoint (50%) of the line connecting the ischial tuberosity and the lateral tibial condyle, with the electrode axis parallel to this line. (2) For the rectus femoris: along the midpoint (50%) of the line connecting the anterior superior iliac spine and the superior border of the patella, with the electrode axis parallel to this line. (3) For the tibialis anterior: along the proximal 1/3 segment of the line connecting the fibular head and the medial malleolus, with the electrode axis parallel to this line. (4) For the lateral gastrocnemius: along the proximal 1/3 segment of the line connecting the calcaneus and the fibular head, with the electrode axis parallel to this line. EMG sensors and wires were secured with elastic bandages.

sEMG data were sampled at 1000 Hz with a 0.5–500 Hz band-pass filter. Offline processing included 4th-order Butterworth 5–150 Hz band-pass filtering to remove motion artifacts and high-frequency noise, followed by rectification using a 100 m root-mean-square (RMS) window. sEMG amplitudes were normalized to peak values during maximal voluntary contraction (MVC), expressed as RMS (%). sEMG values were averaged across each STS trial. The MVC was measured for each muscle: 3 trials of 5-s isometric contraction (e.g., knee extension for rectus femoris) with 1-min rest, normalized to the highest peak amplitude (Winter et al., 1994).

### Statistical analysis

SPSS 20.0 and Graphpad 9.0 software were used for statistical analysis and graphing. Correlations between age, physical activity level, and STS parameters were analyzed using Pearson correlation coefficients (with 95% confidence intervals). Temporal parameters were analyzed using 2 (time: pre-vs post-intervention) × 2 (group: Tai Chi vs control) two-way ANOVA. Sample entropy and EMG data were analyzed using 2 (time) × 2 (group) × 2 (phase: standing vs sitting) three-way ANOVA. Levene’s test confirmed homogeneity of variance, and the Kolmogorov-Smirnov test confirmed normality. For repeated-measures ANOVA, Mauchly’s test assessed sphericity; Greenhouse-Geisser correction was applied if violated. Paired t-tests were used for pre-post comparisons within groups, and independent samples t-tests for between-group comparisons. Bonferroni correction was applied for multiple comparisons. Effect sizes (η_p_^2^ and Cohen’s d) were reported alongside F, t, and corrected *p*-values. Significance was set at α = 0.05. Data are presented as (x̄±s).

## Results

### Age-related changes in parameters and their correlations

**FIGURE 1:**
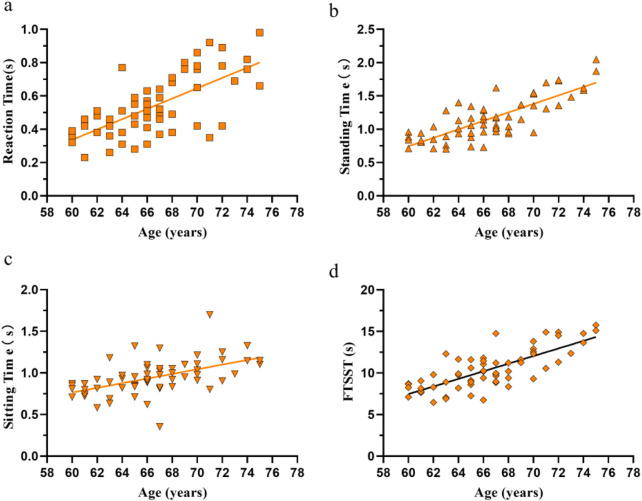
Correlations between age and reaction time **(a)**, standing time **(b)**, sitting time **(c)**, and total time for five sit-to-stand repetitions **(d)** in all participants (n = 60) before the experimental intervention.

Figure 1 showed correlations between age and parameters in all participants before intervention. Age was significantly correlated with reaction time (r = 0.687, 95%CI: [0.524, 0.801], p < 0.0001) (Figure 1a), standing time (r = 0.796, 95%CI: [0.679, 0.873], p < 0.0001) (Figure 1b), sitting time (r = 0.530, 95%CI: [0.319, 0.691], p < 0.0001) (Figure 1c), and total time for FTSST (r = 0.768, 95%CI: [0.638, 0.855], p < 0.0001) (Figure 1d). This indicates that with increasing age, older adults exhibit reduced reaction time and impaired STS ability.

**FIGURE 2:**
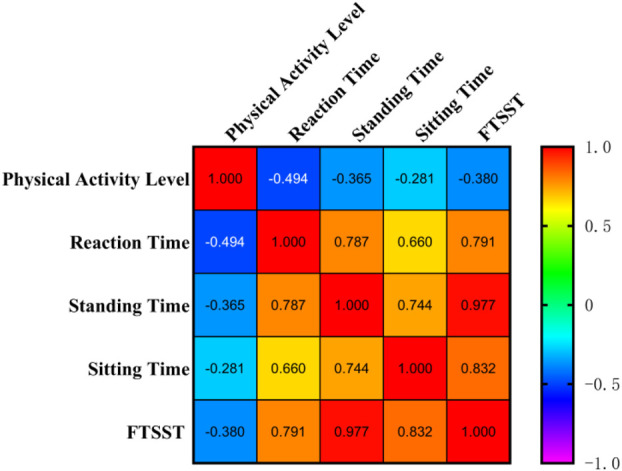
Correlation matrix among physical activity level, reaction time, standing time, sitting time, and total time for FTSST. The numbers in each grid are the correlation coefficients between the two corresponding variables.

Figure 2 showed that physical activity level was significantly negatively correlated with reaction time (r = −0.494, 95%CI: [-0.664, −0.274], *p* = 0.0001), standing time (r = −0.365, 95%CI: [-0.567, −0.123], *p* = 0.004), sitting time (r = −0.281, 95%CI: [-0.500, −0.030], *p* = 0.029), and total time for FTSST (r = −0.380, 95%CI: [-0.578, −0.139], *p* = 0.003). Additionally, reaction time was significantly positively correlated with standing time (r = 0.787, 95%CI: [0.666, 0.868], *p* < 0.0001), sitting time (r = 0.660, 95%CI: [0.487, 0.783], *p* < 0.0001), and total time for FTSST (r = 0.791, 95%CI: [0.672, 0.870], *p* < 0.0001). These findings suggest that lower physical activity is associated with weaker STS performance and longer reaction time (indicating poorer cognitive function) in older adults.

### Temporal parameters during STS

**FIGURE 3:**
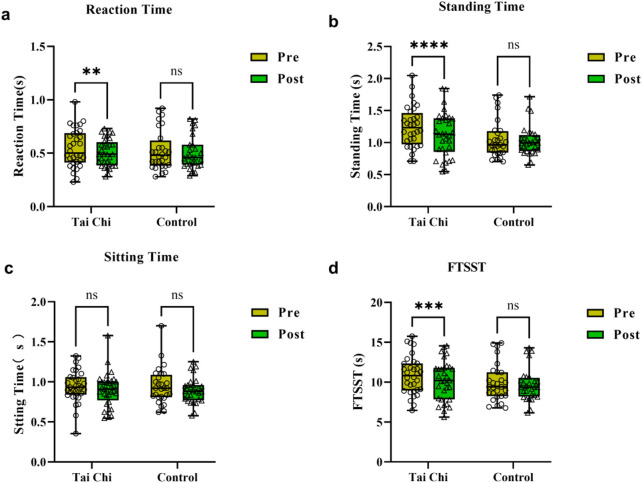
Comparisons of reaction time **(a)**, standing time **(b)**, sitting time **(c)**, and FTSST **(d)** parameters pre and post the experimental intervention in both groups of participants. **, *p* < 0.01; ***, *p* < 0.001; ****, *p* < 0.0001.

Table 2 and Figure 3 showed that the Tai Chi group exhibited significant reductions in reaction time (t = 4.673, *p* < 0.0001, d = 0.829), standing time (t = 5.242, *p* < 0.0001, d = 0.927), and total time for FTSST (t = 4.345, *p* = 0.001, d = 0.768) after 16 weeks of intervention, with no significant change in sitting time. The control group showed non-significant reductions in these parameters. These results indicate that 16-week Tai Chi practice significantly improves cognitive reaction and temporal characteristics during STS in older adults.

**TABLE 2:** Changes in reaction time and STS temporal parameters pre and post intervention (x̄±s).

Measurements	Group	Pre	Post	t values	*p* values
Reaction Time (s)	Tai Chi	0.546 ± 0.2	0.503 ± 0.10^**^	3.637	0.0012
	Control	0.526 ± 0.2	0.505 ± 0.2	1.675	0.198
	t values	0.485	0.028		
	*p* values	>0.999	>0.999		
Standing Time (s)	Tai Chi	1.246 ± 0.3	1.123 ± 0.4^***^	5.242	<0.0001
	Control	1.052 ± 0.3^#^	1.031 ± 0.2	0.8000	0.8005
	t values	2.460	1.171		
	*p* values	0.031	0.488		
Sitting Time (s)	Tai Chi	0.942 ± 0.2	0.901 ± 0.2	1.723	0.1803
	Control	0.948 ± 0.2	0.893 ± 0.2	2.125	0.0756
	t values	0.102	0.151		
	*p* values	>0.9999	>0.9999		
FTSST (s)	Tai Chi	10.858 ± 2.4	10.169 ± 2.5^***^	4.345	0.0001
	Control	9.923 ± 2.4	9.688 ± 2.1	1.390	0.3389
	t values	1.550	0.7992		
	*p* values	0.2464	0.8517		

** and ***, compared with the pre-intervention values, *p* < 0.01, *p* < 0.001; #, compared with the Tai Chi group, *p* < 0.05.

## COP changes during STS

**FIGURE 4:**
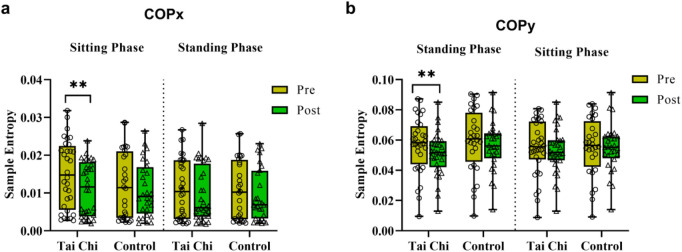
Comparisons of the sample entropy of the COP in x **(a)** and y **(b)** axis during different movement phases pre and post the experimental intervention in both groups of participants. **, *p* < 0.01.

Figure 4 showed the effects of group, time, and STS phase on COP. Significant time effects were observed for both COPx (F (1, 116) = 16.38, *p* < 0.0001, η_p_^2^ = 0.123) and COPy (F (1, 116) = 15.17, *p* = 0.0002, η_p_^2^ = 0.115), with no significant effects of group, phase, or group × time × phase interaction. Further analysis revealed that the Tai Chi group exhibited significantly reduced sample entropy for COPx (0.015 ± 0.008 vs 0.010 ± 0.006, *p* = 0.0041, d = 0.554) and COPy (0.057 ± 0.046 vs 0.051 ± 0.035, *p* = 0.0065, d = 0.615) post-intervention, with no significant changes in the control group. Movement phase (standing/sitting) had no significant effect on COP.

## Lower limb EMG changes during STS

**FIGURE 5:**
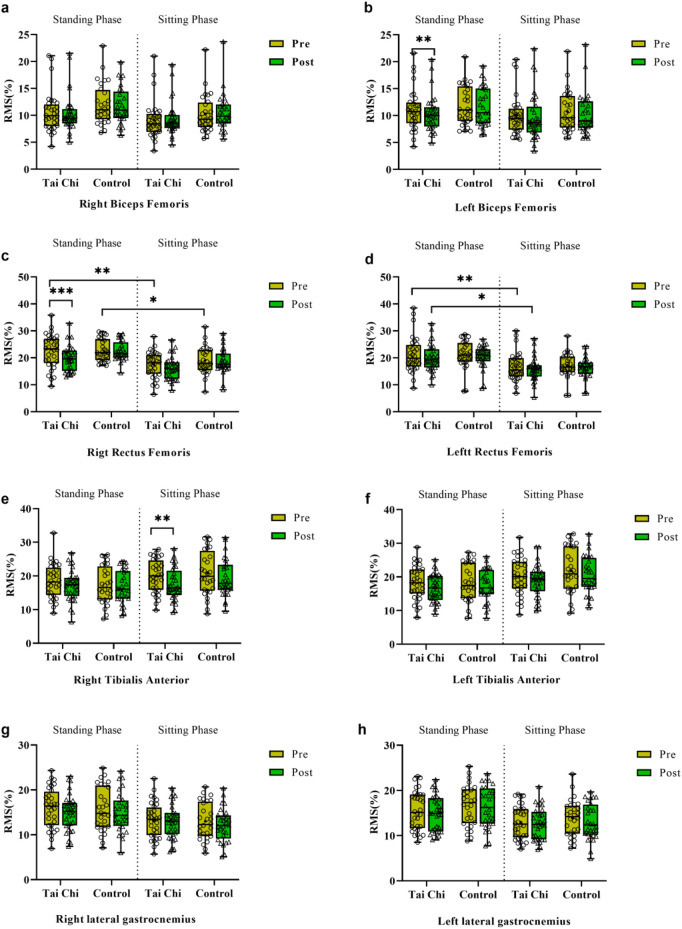
Comparison of EMG activity in lower limb muscles (right biceps femoris **(a)**, left biceps femoris **(b)**, right rectus femoris **(c)**, left rectus femoris **(c)**, right tibialis anterior **(e)**, left tibialis anterior **(f)**, right lateral gastrocnemius **(g)**, left lateral gastrocnemius **(h)**) during different movement phases before and after the experimental intervention in both groups of participants. *, *p* < 0.05; **, *p* < 0.01; ***, *p* < 0.001.

For the biceps femoris, the experimental group showed a significant reduction in RMS (%) of the left biceps femoris during the standing phase after 16-week Tai Chi intervention (11.08% ± 3.81% vs 10.33% ± 3.38%, t = 3.840, *p* = 0.0056, d = 0.678), with a non-significant decrease in the right biceps femoris. For the rectus femoris, changes were more complex. Significant phase effects were observed: RMS (%) values during sitting were significantly lower than during standing for both left and right rectus femoris (Figures 5c,d). Additionally, the experimental group exhibited a significant reduction in RMS (%) of the right rectus femoris during standing after intervention (22.50% ± 6.18% vs 19.91% ± 4.88%, t = 4.842, *p* = 0.0001, d = 0.857). For the tibialis anterior, only the Tai Chi group showed a significant reduction in RMS (%) of the right tibialis anterior during sitting after intervention (19.99% ± 4.96% vs 17.58% ± 4.81%, t = 4.142, *p* = 0.0018, d = 0.732). No significant changes were observed for the gastrocnemius.

## Discussion

In this randomized controlled trial that integrated phase segmentation, nonlinear center of pressure (COP) sample entropy, and multi-muscle surface electromyography (sEMG), we demonstrated that 16 weeks of Yang-style Tai Chi training led to clinically meaningful and mechanistically interpretable improvements in sit-to-stand (STS) performance among community-dwelling older adults. Tai Chi significantly reduced the time required to complete the Five Times Sit-to-Stand Test (5-STS). This functional improvement was accompanied by a reduction in mediolateral COP sample entropy and selective decreases in sEMG amplitude of the biceps femoris, rectus femoris, and tibialis anterior muscles—collectively suggesting a shift toward more efficient neuromuscular control. To our knowledge, this is the first RCT to elucidate neuromuscular adaptations underpinning Tai-Chi-mediated improvements in STS performance. This study validates Tai Chi’s effects on STS ability using multi-dimensional indices (temporal parameters, COP trajectory, and sEMG signals), providing scientific evidence for promoting Tai Chi in geriatric rehabilitation.

In this study, a significant positive correlation was observed between age and STS duration, consistent with existing literature. A meta-analysis by Xue et al. (2021) reported that STS time increases with age, averaging 0.3 s per year, with an accelerated decline in adults over 70 years old. Yamada and Demura (2009) found that quadriceps cross-sectional area decreases annually in 60–75 years old, directly impairing lower limb muscle power during STS. In the present study, age was moderately negatively correlated with physical activity level (r = −0.496), and physical activity level was weakly negatively correlated with FTSST (r = −0.38), suggesting that age-related reduction in physical activity further compromises the ability to perform repeated STS transitions. Wolfson et al. (1996) demonstrated that older adults with high physical activity levels have higher mitochondrial ATP production rates than sedentary individuals, providing a metabolic explanation for the association between physical activity and STS capacity. Additionally, reaction time was moderately positively correlated with age (r = 0.687), reflecting a general slowing of motor responses in older adults. This aligns with findings by Singh and Natsume (2023), who reported that older adults exhibit prolonged premotor potential latency in the prefrontal cortex during STS, possibly due to reduced dopaminergic neuron density in the basal ganglia and impaired white matter integrity between the prefrontal cortex and motor cortex.

In this study, the Tai Chi group showed significant improvements in reaction time and STS duration, consistent with earlier findings. Wayne et al. (2014) suggested that long-term Tai Chi practice may increase brain volume and enhance neural connectivity, potentially underlying improvements in cognitive function and reaction speed. Notably, sitting time showed no significant change, supporting the hypothesis that age-related declines in eccentric muscle control precede other functional losses (Choi, 2016), as eccentric control (critical for sitting) requires longer training to improve.

The Tai Chi group also exhibited significantly reduced sample entropy for both anteroposterior (COPx) and mediolateral (COPy) after 16 weeks, while no changes were observed in the control group, and no significant phase-specific effects were detected. Our data showed reduced COP entropy post-training, indicating reduced postural variability. This confirms that functional STS gains are paired with improved balance control, a key fall-risk protector. COP trajectory sample entropy is a key indicator of balance control. Reduced COP sample entropy reflects more regular and stable postural control during dynamic tasks (Montesinos et al., 2018; Fischer et al., 2023). Tai Chi emphasizes whole-body coordination and sequential movement initiation from the waist, which may enhance neuromuscular efficiency and optimize balance control during STS. These results align with Tsang and Hui-Chan (Tsang and Hui-Chan, 2004), who observed reduced COP complexity during standing after 4 weeks of Tai Chi training, possibly due to enhanced ankle-hip coordination and reduced compensatory sway. Additionally, Tai Chi has been shown to improve dynamic stability and gait control (Chen et al., 2024; Zhang et al., 2008; Tsang and Hui-Chan, 2004), as well as muscle strength and flexibility (Bai et al., 2024), collectively contributing to reduced fall risk.

Regarding lower limb neuromuscular control strategies, we found that EMG amplitude of the rectus femoris was lower during the sitting phase compared to the standing phase. This is consistent with the phenomenon that eccentric contractions (dominant during sitting) produce lower EMG amplitudes than concentric contractions (dominant during standing) (Pasquet et al., 2000). After 16-week Tai Chi intervention, the experimental group showed reduced EMG amplitudes of the left biceps femoris and right rectus femoris during standing, possibly reflecting improved neuromuscular efficiency. Tai Chi’s emphasis on center-of-mass transfer and coordination requires precise bilateral muscle control, which may optimize motor unit recruitment during STS. Reduced EMG amplitude of the rectus femoris (standing phase) and right tibialis anterior (sitting phase) may reflect enhanced eccentric control of ankle dorsiflexion, likely due to Tai Chi’s emphasis on slow and controlled lower limb movements (Wu et al., 2004). Combined with reduced COP sample entropy, these findings indicate improved muscle activation economy, potentially due to refined motor unit recruitment by the central nervous system (Li et al., 2008).

## Conclusion

This study demonstrated that aging is associated with declines in reaction time and STS ability in older adults. A 16-week randomized controlled trial confirmed that Tai Chi intervention significantly improves STS performance, accompanied by adaptive changes in lower limb neuromuscular electrophysiology. This study is the first to validate Tai Chi’s effects on STS ability using multi-dimensional indices (temporal parameters, COP trajectory, and EMG signals), providing scientific evidence for promoting Tai Chi in geriatric rehabilitation.

## Limitations of study

In this study there were four limitations. (1) Small sample size may reduce statistical power for subgroup analysis; (2) lack of neuroimaging (e.g., EEG, fNIRS); to explore cortical activation during STS; (3) no prospective fall incidence tracking. While STS is a validated fall risk marker (Zhang et al., 2013), we could not confirm whether Tai Chi-mediated STS improvements translate to reduced fall rates; (4) Another key limitation is the passive control group (habitual lifestyle) rather than an active comparator (e.g., supervised stretching or walking). This prevents us from distinguishing whether STS improvements are specific to Tai Chi’s unique movements (e.g., center-of-mass transfer, slow eccentric contractions) or simply due to supervised exercise exposure. Future studies should include larger samples, longer follow-ups to assess long-term effects (fall incidence tracking), and integrate fNIRS/EEG to investigate neural mechanisms underlying Tai Chi’s benefits. Additionally, future RCTs should include an active control to clarify Tai Chi’s specific benefits.

## Data Availability

The raw data supporting the conclusions of this article will be made available by the authors, without undue reservation.
